# Digitally Quantifying Growth and Verdancy of *Lolium* Plants In Vitro

**DOI:** 10.3390/plants14101499

**Published:** 2025-05-16

**Authors:** Mara B. Depetris, Adam M. Dimech, Kathryn M. Guthridge

**Affiliations:** Agriculture Victoria Research, Department of Energy, Environment and Climate Action, Bundoora, VIC 3083, Australia; maradepetris@gmail.com (M.B.D.); adam.dimech@agriculture.vic.gov.au (A.M.D.)

**Keywords:** grasses, ryegrass, fescue, plant growth regulators, tissue culture, image analysis

## Abstract

The image analysis of plants provides an opportunity to measure changes in growth and physiology quantitatively, and non-destructively, over time providing significant advantages over traditional methods of assessment which often rely on qualitative and subjective measures to distinguish between different treatments or genotypes in an experiment. Image analysis techniques are commonly deployed for the analysis of plants in the field or glasshouse, but few studies have demonstrated the use of image analysis to phenotype plants grown under aseptic conditions in culture media. *Lolium* × *hybridum* Hausskn ‘Shogun’ plants were germinated in vitro and cultured on media containing combinations of thidiazuron [1-phenyl-3-(1,2,3-thiadiazol-5-yl) urea] (TDZ), N6-benzylaminopurine (BA) and gibberellic acid (GA3) or on phytohormone-free control media. RGB images were taken of the plants throughout the experiment and morphological image analysis techniques were used to quantify changes in plant development. A novel approach to quantitatively measure ’greenness‘ in plants using the CIELAB colour model (L*a*b) colour space from RGB images was developed. This methodology could be utilised to develop improved in vitro growth protocols for *Lolium* and grass species with similar morphology.

## 1. Introduction

A common challenge in tissue culture experiments is the relative difficulty in collecting quantitative growth data for the purposes of demonstrating the efficacy of treatments. The use of non-destructive image analysis to quantify growth and morphological changes in plants in vitro is a long-established technique [1], although few studies have utilised it. Recent improvements in imaging technology and the availability of open-source computer vision software have made this technique more accessible. Programs such as PlantCV [2], which was originally designed to facilitate the analysis of images derived from glasshouse-based automated plant phenotyping systems, can also be adapted to analysing image data sets collected from other systems or set-ups if a suitable image analysis pipeline is written [3,4,5].

Image analysis techniques permit the replacement of manually derived qualitative or semi-quantitative measurements with quantitative measures that improve accuracy and remove subjectivity from the data collection process. One of the challenges for image analysis with tissue-cultured plants has been obtaining sufficiently clear images of the plants without breaking sterility or suffering occlusion from condensation on the inside of the culture vessel [6,7,8]. The development of image capture and analysis systems that accommodate these requirements is critical to the successful phenotyping of plants grown in vitro.

*Lolium* is a genus of cool-season grasses, which includes perennial ryegrass (*Lolium perenne* L.) and tall fescue (*Lolium arundinaceum* L.) that are utilised in pasture-based agriculture globally because they are a nutritious and palatable forage crop [9]. Commercial perennial *Lolium* species have a productive and persistent extended production period that reduces the annual cost for pasture renovation, growing well under a range of environmental conditions and exhibiting superior tolerance to grazing pressure [10].

*Lolium* species are propagated using tissue culture for the purposes of regeneration [11], transformation [12], in vitro flowering [13] and clonal propagation. The application of plant growth regulators to tissue culture media is important for influencing the growth and morphology of plants in vitro. Auxins exert a strong influence over physiological processes including the initiation of cell division, DNA replication, organisation of meristems, vascular differentiation, apical dominance, root formation, delayed leaf senescence and fruit ripening [14]. Cytokinins stimulate cell division, induce adventitious bud formation, release lateral bud dormancy, promote lateral bud growth, leaf expansion, chlorophyll synthesis, chloroplast development and delay leaf senescence [15]. Gibberellins (GAs) influence the formation of embryos, roots and shoots, seed germination, stem elongation (cell division and elongation) and floral initiation [16].

The absolute concentration of growth regulators and the relative concentration of one class with another are important influences of growth and physiological development. Previous studies can be used to guide the development of optimal media for tissue culture experiments. For instance, N^6^-benzylaminopurine (BA, a synthetic cytokinin) has been used at a concentration of 8.8 µM in Poaceae species like *Zea mays* [17], *Pennisetum glaucum* [18] and *Bambusa arundinacea* for shoot multiplication [19]. Thidiazuron, a synthetic cytokinin-like plant growth regulator used to induce the regeneration of plants in tissue culture, was used in *L. perenne* to induce in vitro flowering at a concentration of 9 µM [13].

Gibberellic acid (GA_3_) has been used at a concentration of 1 µM in monocots such as *L. temulentum* [20,21,22], *B. arundinacea*, *Dendrocalamus brandisii* [23] and at a concentration of 1.43 µM in *Bambusa vulgaris*, *D. giganteus* and *D. strictus* [24]. GA_3_ is also commonly used in dicotyledonous species such as *Malus domestica* to increase the elongation of adventitious shoots [19], in *Ceropegia bulbosa* to produce in vitro flowering [25] and in *Cephaelis ipecacuanha* for shoot multiplication [26].

Whilst the impact of plant growth regulators can often be seen with the eye, the challenge for growers is in being able to accurately quantify changes in physiology, morphology and health. In this study, image analysis technology was developed to quantitatively measure plant growth and morphology in tissue culture, using the application of various phytohormone treatments to introduce a range of phenotypes. These phenotypes were used to test the image analysis pipeline and validate the results. Conventional morphological analyses were performed, such as area, convex hull area, perimeter and solidity, whilst a novel measure of ‘greenness’ was developed. These were used to quantify changes in growth and morphology over the duration of the study.

## 2. Results

### 2.1. Greenness Index

**Figure 1:**
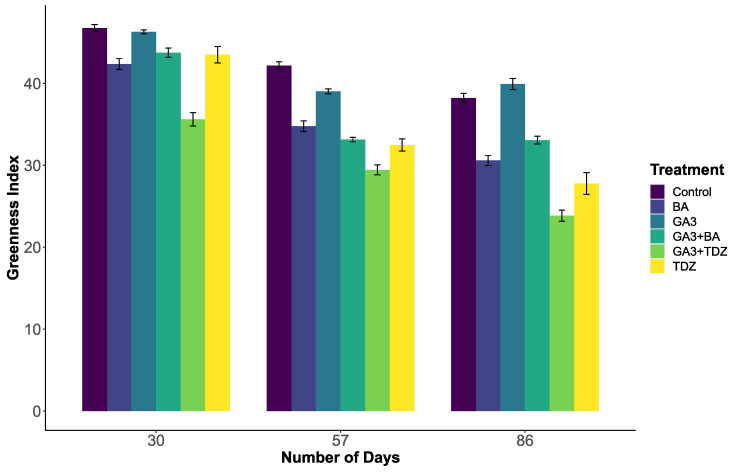
Mean greenness values for ryegrass plants growing in vitro at Days 30, 57 and 86. Error bars represent standard error to a 95% confidence interval.

The process for measuring ‘greenness’ accurately detected differences in verdancy (Figure 1) between different treatment groups over the course of the experiment. Throughout the experiment, greenness declined over time. The greatest mean greenness index scores were observed in the GA_3_ and Control treatments (46.3 and 46.8, respectively, Table S1).

The amount of variance explained by the linear mixed-effects model for greenness was 69%. Within this model, the greenness scores were found to be significantly affected by day, treatment and their interaction (Table 1).

### 2.2. Area

**Figure 2:**
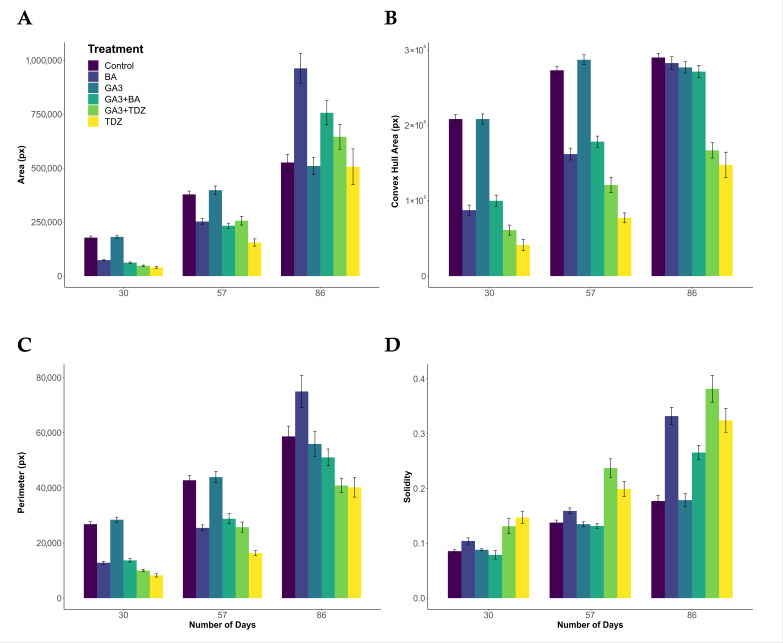
(**A**) Mean area values for ryegrass plants growing in vitro at Days 30, 57 and 86; (**B**) mean convex hull area for ryegrass plants growing in vitro at Days 30, 57 and 86; (**C**) mean perimeter values for ryegrass plants growing in vitro at Days 30, 57 and 86; (**D**) mean solidity values for ryegrass plants growing in vitro at Days 30, 57 and 86. Error bars represent standard error to a 95% confidence interval.

Plant growth, as measured by the pixel area, was observed to increase throughout the experiment (Figure 2A); however, the results were variable. At Day 30, pixel area was the greatest in the Control (178,288.8 px) and GA_3_ (181,651.2 px) treatment groups. The trend continued to Day 57, but by Day 86, the area was greater (962,570.5 px) in the BA plants group. The amount of variance explained by the linear mixed-effects model for area was 62%. Within this model, the area measurements were found to be significantly affected by day, treatment and their interaction (Table 1).

### 2.3. Convex Hull Area

Convex hull area, a measure of the architecture and size of a plant, increased in most groups for the duration of the experiment (Figure 2B). The exception to this was the Control and GA_3_ treatments which grew to such an extent by Day 57 that growth was constrained by the vessel. From Days 30 to 57, the greatest convex hull area was observed in the Control and GA_3_ treatment groups. At Day 86, the BA and GA_3_ + BA treatments had increased so that there was little significant difference between them and the Control and GA_3_ groups. Throughout the experiment, the smallest convex hull area was observed in the TDZ treatment. The amount of variance explained by the linear mixed-effects model for the convex hull area was 73%. Within this model, the convex hull area measurements were found to be significantly affected by day, treatment and their interaction (Table 1).

### 2.4. Perimeter

Perimeter was highly variable across the experiment (Figure 2C). At Days 30 and 57, the greatest perimeter measurements were observed in the Control and GA_3_ treatment groups. By Day 86, the greatest perimeter measurement was in the BA treatment (74,951.2 px). The amount of variance explained by the linear mixed-effects model for the perimeter was 56%. Within this model, the perimeter measurements were found to be significantly affected by day, treatment and their interaction (Table 1).

### 2.5. Solidity

Solidity, being the ratio of area to convex hull area, increased to varying degrees throughout the experiment (Figure 2D). This measurement considers the ‘bushiness’ of plants, irrespective of their size, compared to absolute measurements of the convex hull area, which is partially a product of size. Solidity (‘bushiness’) was greatest in the GA_3_ + TDZ and TDZ treatment groups, which were observed to have numerous small tillers in comparison to many of the other treatments where the tillers were fewer but larger. At the conclusion of the experiment, the greatest solidity was observed in the GA_3_ + TDZ treatment (0.381), followed by TDZ (0.324). The lowest solidity was recorded for the Control (0.177) and GA_3_ (0.179) treatments. The amount of variance explained by the linear mixed-effects model for solidity was 59%. Within this model, the solidity measurements were found to be significantly affected by day, treatment and their interaction (Table 1).

### 2.6. General Survival

Some minor losses were observed during the experiment. All 15 plants in the GA_3_ + BA and GA_3_ treatment groups survived the duration of the experiment. A total of 87% of plants survived in the Control treatment, 80% in the BA treatment, 73% in the GA_3_ + TDZ treatment and 60% in the TDZ treatment group. Of these, contamination caused the loss of a single plant on each of the BA and TDZ treatments on Day 57 and for BA and TDZ treatments on Day 86; additionally, two plants were lost on Day 57 for GA_3_ + TDZ and Day 86 for Control. All other losses were due to plant death. Summary data for plant survival and all other measurements are provided in Table S1.

## 3. Discussion

This research demonstrated a novel method for measuring greenness in aseptically cultured plants using RGB imaging. The greenness of a leaf is most strongly correlated with chlorophyll content, but its perception is also affected in vitro by the presence or absence of other leaf pigments and can be negatively affected by changes in nutrient status, the availability of carbohydrates in culture media, levels of ambient light and the presence and ratio of plant growth regulators [27,28]. These conditions can decrease the chlorophyll content reducing the greenness, light absorption, photosynthetic activity, plant yield and biomass production [29]. Mean greenness declined in all treatments over time, which was expected due to the depletion of carbohydrates in the culture media, as well as senescence associated with maturity. 

This study demonstrated that greenness was also affected by plant growth regulators. Both the absolute concentration of plant growth regulators and their relative concentrations to each other will elicit different physiological responses in vitro. In general, the concentration of PGR declines over time due to temperature and light degradation [30] as well as metabolism. The greenest ryegrass plants were grown either in the absence of PGRs or the absence of BA and TDZ. The presence of TDZ in culture media led to a greater yellowing of plants than the BA treatment, which may be a result of the greater physiological impact of TDZ on plant cells relative to BA [31]. The use of image analysis to quantitatively measure plant growth and development in vitro is relatively uncommon despite the growth of image-based phenotypic analysis for glasshouse- and field-grown plants. One of the challenges to overcome with image-based analyses in tissue culture systems is the problem of distortion caused by the design and material composition of culture vessels or occlusion due to condensation [6,7,8]. Whilst removing the lids from vessels provides an unhindered view of plants, it also results in the loss of aseptic status and would likely lead to contamination and eventual loss of the plant (and is thus considered a destructive technique). The most comprehensive system to overcome the challenges of in vitro image analysis has been developed by Bethge [32], who utilised a range of sensors including a laser distance sensor, RGB camera, micro spectrometer and thermal camera to collect highly accurate multi-sensor image data of several species growing in standard polycarbonate culture tubes and Petri dishes. The combination of sensors utilised by Bethge [32] permitted the accurate measurement of explant height, chlorophyll fluorescence and growth. Their use of a laser distance scanner was necessitated by their desire to obtain explant height measurements from overhead images. Mestre [8] used RGB and NIR imagery to measure the growth of *Nandina* from overhead images, whilst Dhondt [6] and Faragó [7] measured the growth of *Arabidopsis* from above. The methodology presented in this paper utilised RGB side-view imagery to achieve accurate measurements of growth in ryegrass without the complex set-ups utilised by Bethge [32] and Dhondt [6].

The use of image-based analysis to quantify the growth of ryegrass in vitro was highly successful. Isolating plants from the background was relatively straightforward and did not require the use of advanced thresholding techniques, of which there are many [33]. This study was able to distinguish between treatment groups based on differences in area, convex hull area, solidity and perimeter over time. This was possible using a customised photography set-up that ensured images were collected consistently throughout the experiment and open-source software that streamlined the process of image analysis. This study utilised PlantCV [34], which has also been utilised by other researchers analysing tissue-cultured plants [32], although software such as MATLAB [7], ImageJ [35] could also be used [6].

The ability to quantitatively measure plant area, convex hull area, solidity and perimeter means that better-quality data are available to guide decision-making in terms of optimising culture media for particular purposes. For instance, the results of this study show that 8.8 μmol L^−1^ BA led to significant increases in the area of the plant, which could be used as a proxy for biomass. This relationship has been demonstrated in numerous other image analysis systems [32].

Convex hull area is an informative measurement, and in this study, it was observed that the greatest convex hull area was initially observed in the Control treatments or plants grown on 2.8 μmol L^−1^ GA_3_. Convex hull area reflects the architecture and the size of the plants. However, as the explants grew, some reached the limit of the culture vessel. Nevertheless, we could see a clear trend where the convex hull area of the Control and BA treatments exceeded all others, which was reflected in an open architecture. A better measure of ‘bushiness’ (plant density) can be obtained from the measurements of solidity, which is the ratio of area to convex hull area. This measurement takes the size of the plant into account. So, it was observed that the greatest values for solidity were in the GA_3_ + TDZ, BA and TDZ treatment groups, which was reflected in their relative density when compared to the Control and GA_3_ treatments in particular. This bushiness was due to the proliferation of tillers in each of these treatments, an observation seen in other related crops [36].

The methodology described in this paper could be readily applied to other species grown in vitro, but imaging pipelines would require optimisation for each species owing to different growth habits and colour profiles. Adjustments to thresholding values and other parameters may be required to accommodate differences in imaging conditions introduced by the use of alternative culture vessels, light sources and/or backgrounds. Automated methods, such as the application of an Otsu process [37], could assist in this, although these were not found to be helpful with *Lolium*. We also note that the presence of other pigments, such as anthocyanins or carotenoids, could introduce bias in accurately determining verdancy [38,39,40], where an assumption is made that greenness directly correlates with chlorophyll content.

This research demonstrates the feasibility of using common imaging tools and open-source software to accurately measure changes in plant physiology and morphology over time, as demonstrated in ryegrass subjected to different plant growth regulator treatments. Images were collected and measurements taken without risk to the sterility of the culture vessels, providing accurate quantitative phenotypic data over time.

## 4. Materials and Methods

### 4.1. Plant Material

Seeds from *Lolium × hybridum* ‘Shogun’, a late-flowering tetraploid interspecific hybrid between *Lolium perenne* ‘Bealey’ and *Lolium multiflorum* ‘FSTII’, were purchased from Hamilton Produce (Hamilton, Victoria, Australia) in April 2022 and stored at 4 °C in the dark until required.

### 4.2. Seed Sterilisation

Ryegrass seeds were surface sterilised in a 50 mL centrifuge tube containing 10 µL of Tween 20^®^ and 40 mL of sodium hypochlorite (4% *m*/*v*), shaken for 1.5 h. The seeds were then rinsed thoroughly with sterile water 7–8 times, before being left to soak in deionised water in centrifuge tubes for 20–30 min. Several more water rinses were applied before the seeds were dried on sterilised filter paper.

### 4.3. Seed Germination

SteriCon-13™ 473 mL gamma-irradiated culture vessels (Catalogue C1932, PhytoTech Labs, Lenexa, KS, USA) were filled with 110 mL of sterilised culture media containing water plant agar (20 g L^−1^) and cefotaxime (5.5 µM). The seeds were arranged with the embryo facing down in three rows with three seeds per row. A total of 20 tubs were used for the germination process, incubated at 25 ± 2 °C under cool, white, TL-D 58W/840 1SL/25 fluorescent tubes (Philips, North Ryde, New South Wales, Australia) and incandescent globes (Catalogue AC40CSES, Crompton Lamps, Bradford, UK) with a light intensity of 110 µmol m^−2^ s^−1^, set at a 16 h photoperiod for 7 days.

### 4.4. Hormone Treatments

Seedlings were subcultured onto 5 different media using 15 replicates per treatment. Murashige and Skoog [41] medium (MS) was supplemented with B5 vitamins [42], 30 g L^−1^ sucrose and 3 g L^−1^ Phytagel. Table 2 describes the components of each medium. Plant growth regulator concentrations were selected based on previous studies of Poaceae (grass) species [17,18,25,43,44]. The control media contained no plant growth regulators.

**Figure 3:**
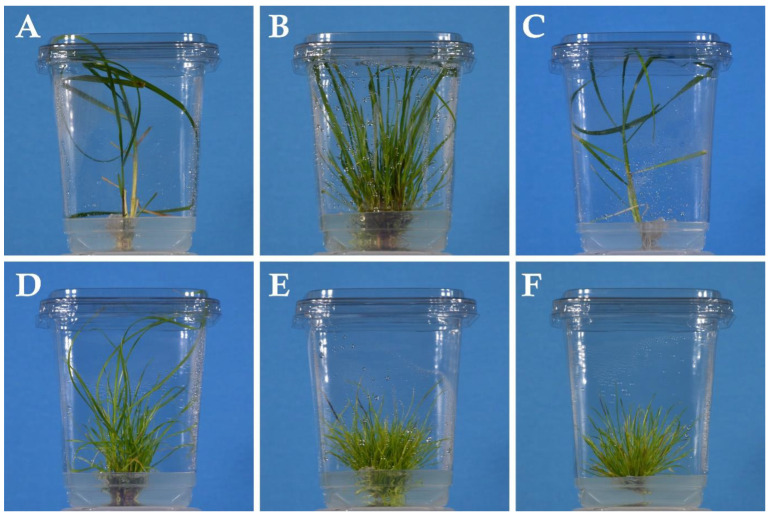
Representative images of *Lolium* plants growing in vitro at Day 86 of the experiment: (**A**) Control; (**B**) BA; (**C**) GA_3_; (**D**) GA_3_ + BA; (**E**) GA_3_ + TDZ; (**F**) TDZ.

Cultures were maintained under the same environmental conditions as the germination step. The plants were transferred on to fresh media every 4 weeks for 4 months (Figure 3).

### 4.5. Collection of Images

**Figure 4:**
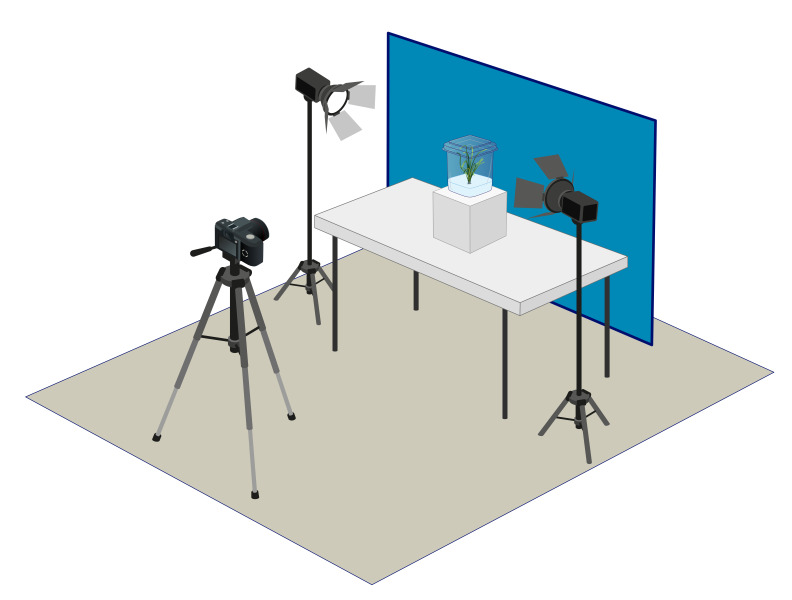
Schematic diagram of imaging station used to collect images of tissue culture vessels for this experiment.

Photographs were taken of the tissue-cultured plants on Days 30, 57 and 86 of the experiment using a Nikon D5500 Digital Single-Lens Reflex (dSLR) camera (Nikon Corporation, Shinagawa, Tokyo, Japan) mounted on a tripod with focal length of 140 mm, aperture f/13, exposure 1/25 sec and ISO 100. RGB images were saved in JPEG format with dimensions of 6000 × 4000 pixels. A custom-made imaging station was established with a blue background designed to facilitate the efficient thresholding of images (Figure 4).

### 4.6. Image Analysis

**Figure 5:**
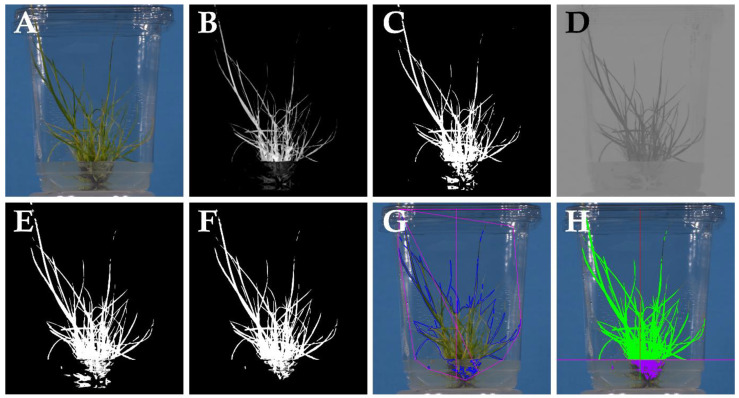
A summary of the steps in the image analysis pipeline used to identify ryegrass plants in vitro: (**A**) original RGB image; (**B**) conversion of the RGB image into CMYK with the Y-channel selected; (**C**) binarisation of the Y-channel image; (**D**) conversion of the RGB image into L*a*b with the *a channel selected; (**E**) binarisation of the *a-channel; (**F**) logical-OR operation to combine Y-channel and *a-channel binary masks; (**G**) morphological analysis using PlantCV; (**H**) measurement of foreground area above and below the medium level.

A customised PlantCV [34] image analysis pipeline (Figure 5) was written to perform the image analysis steps. PlantCV version 3.12.0 running with OpenCV version 3.4.10.35 and Python version 3.8.2 under CentOS Linux version 7.9.2009 operating system was used on the Biosciences Advanced Scientific Computer (BASC) at the Centre for AgriBioscience in Bundoora, Victoria, Australia. RGB images were loaded into the pipeline and cropped to a dimension of 2800 × 2600 pixels. RGB images were converted into the CMYK colour space (cyan, magenta, yellow and black bands; Figure 5A). The Y-channel was found to be useful in separating the plant from background (Figure 5B) and was binarised with a threshold of 45 (Figure 5C); this value was identified during the process of code optimisation. A 5 × 5 dilation step was followed by an erosion step before a 3 × 3 cruciform kernel was used to perform an area opening. To aid in the isolation of plant material that was partially obscured by culture media, a separate process was initiated to convert the RGB images into the L*a*b colour space [45] (Figure 5D). The *a channel was binarised with a threshold of 124 (Figure 5E). The binary image was inverted and a fill operation performed. A binary mask was generated by performing a logical-OR operation with the binarised Y and *a images (Figure 5F) to combine the two plant masks together. The binary mask was then used to isolate plants from background (Figure 5G). Generic PlantCV visible spectrum analysis processes were then deployed to identify objects and perform morphological analyses (Figure 5H).

**Figure 6:**
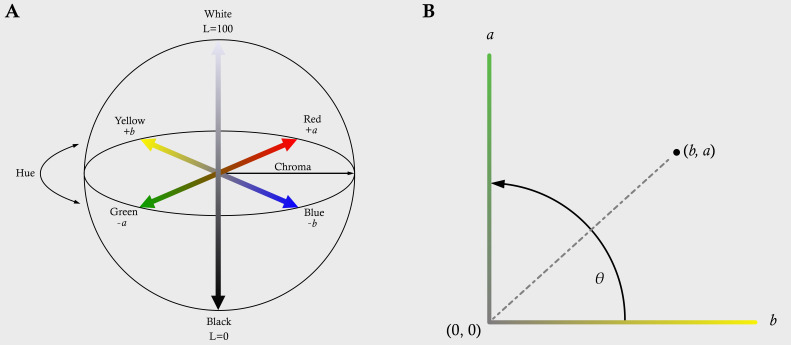
(**A**) The CIELAB colour space diagram. The L*a*b, colour system represents quantitative relationship of colours on three axes: *L* value indicates lightness, and *a* and *b* are chromaticity coordinates. (**B**) The mean values of *a* (where *a* < 0) and *b* (where *b* > 0) can be used to generate a (*b*, *a*) co-ordinate. The angle created between (*b*, *a*) and (0, 0) can be used to create an index of “greenness” by rescaling it from 0 (*θ =* 0°) to 100 (*θ =* 90°), where *θ* increases with increasing verdancy.

In addition, a novel “greenness” index was developed using the following procedure: RGB images were converted into the L*a*b colour space and non-zero values saved into an array that contained the *L*, *a* and *b* values for every foreground (plant) pixel if *a* < 0 and *b* > 0. This was to collect green (‘verdancy’) values from the *a* (Green–Red) channel and yellow (‘chlorosis’) values from the *b* (Blue–Yellow) channel. To calculate a “greenness” score, the absolute of the mean value of *a* and *b* were calculated for each image. Since *a* and *b* values are at right-angles in the L*a*b colour model [45](Figure 6A), their combination could be plotted as a co-ordinate on a right-angle axis where *a* equalled *y* and *b* equalled *x* (Figure 6B). The angle between (*b*, *a*) and (0, 0), designated as *θ*, was calculated according to the following equation:

$$\theta =\left|arctan\left(\frac{a}{b}\right)\times \left(\frac{180}{\pi }\right)\right|$$

Values of *θ* were rescaled between 0 and 100 to give a “greenness” index, where a low score indicated more chlorosis and a higher score indicated more verdancy.

### 4.7. Data Analysis

Image data were analysed in R v.4.3.1 [46] using RStudio v.2023.06.1, build 524. Mean values for greenness, pixel area, convex hull area, solidity and perimeter were calculated.

To model greenness, pixel area, convex hull area, solidity and perimeter, the following linear mixed-effects model in R notation was used:

response ~ date + treatment + date:treatment + (1|plant_id)

where date is a factor with 3 levels, treatment is a factor with 6 levels, date:treatment is a factor with 18 levels and plant_id is a factor with 90 levels. The expression (1|plant_id) specifies an independent random intercept to account for repeat readings of the plants in each cultivar. Statistical significance was indicated where *p* < 0.05. Compact letter displays were computed via a calculation of Estimated Marginal Means from the Generalised Linear Model (GLM) via the emmeans [47] and multcomp [48] packages in R.

## 5. Conclusions

This research demonstrates the feasibility of using digital image analysis to accurately measure changes in plants grown in tissue culture vessels. Images were collected and measurements were taken without risk to the sterility of the cultures, providing accurate quantitative phenotypic data over time. A novel “greenness” index, which accurately quantified verdancy, was developed.

## Figures and Tables

**Table 1:** Summary of analysis of variance (ANOVA, Type II Wald chi-square test) results. *q*-values are Holm multiple test-corrected *p*-values.

Response Variable	R^2^	Factor	χ^2^	DF	Pr (>χ^2^)	*q*-Value
Area	0.62	day	310.412	2	<0.0001	<0.0001
		treatment	15.873	5	0.007	0.007
		day:treatment	55.043	10	<0.0001	<0.0001
Convex Hull Area	0.73	day	233.200	2	<0.0001	<0.0001
		treatment	363.638	5	<0.0001	<0.0001
		day:treatment	46.099	10	<0.0001	<0.0001
Greenness	0.69	day	289.825	2	<0.0001	<0.0001
		treatment	255.439	5	<0.0001	<0.0001
		day:treatment	30.877	10	0.0006	0.0019
Perimeter	0.56	day	217.197	2	<0.0001	<0.0001
		treatment	58.236	5	<0.0001	<0.0001
		day:treatment	30.118	10	0.0008	0.0018
Solidity	0.59	day	235.852	2	<0.0001	<0.0001
		treatment	87.632	5	<0.0001	<0.0001
		day:treatment	37.406	10	<0.0001	0.0002

**Table 2:** Plant growth regulators and their concentrations used throughout this experiment.

Designation	Plant Growth Regulator(s)	Concentration (µM)
Control	NA	0
BA	N6-benzylaminopurine	8.8
GA_3_	Gibberellic acid	2.8
TDZ	Thidiazuron	9
GA_3_ + TDZ	Gibberellic acid	2.8
	Thidiazuron	9
GA_3_ + BA	Gibberellic acid	2.8
	N6-benzylaminopurine	8.8

## Data Availability

Data are contained within the article and Supplementary Materials section.

## Supplementary Materials

The following supporting information can be downloaded at https://www.mdpi.com/article/10.3390/plants14101499/s1: Table S1: Mean measurements for greenness, area, convex hull area, solidity and perimeter for ryegrass plants growing at Days 30, 57 and 86.
