# Supplementary material for: Digitally Quantifying Growth and Verdancy of Lolium Plants In Vitro
Source: Plants (Basel). 2025 May 16;14(10):1499. doi: 10.3390/plants14101499 (PMC12115340; doi:10.3390/plants14101499)
Supplement: Supplementary file 1 [file plants-14-01499-s001.zip › plants-3601220-supplementary.pdf]

Supplementary Materials

Table S1. Mean measurements for greenness, area, convex hull area, solidity and perimeter for perennial ryegrass plants growing at days 30, 57 and 86, with standard error (to 95% confidence interval) indicated. Values within a column with different letters were significantly different ($p<0.05$) based on differences in estimated marginal means for general linear models of each parameter. The loss of plants due to death or contamination is reflected in the value for *n*.

Day	n	Treatment	Mean Greenness ± SE (units)	Mean Area ± SE (pixels)	Mean Convex Hull ± SE (pixels)	Mean Solidity ± SE (units)	Mean Perimeter ± SE (pixels)
30	15	Control	46.8±0.4^{a}	178288.8±7377.2^{d}	2084913.3±59466.8^{fg}	0.086±0.003^{ab}	26822.8±878.8^{cdef}
	15	BA	42.4±0.7^{ab}	73941.0±3133.7^{bc}	875444.5±67420.2^{abc}	0.104±0.006^{abc}	12830.6±528.8^{ab}
	15	GA_3	46.3±0.2^{a}	181651.2±5848.3^{d}	2085422.8±66207.0^{fg}	0.088±0.002^{ab}	28435.6±925.3^{def}
	15	GA_3+BA	43.8±0.6^{ab}	62321.5±3004.1^{b}	999338.0±75748.8^{abcd}	0.079±0.008^{a}	13700.8±615.3^{b}
	15	GA_3+TDZ	35.6±0.8^{cde}	47788.5±3314.4^{ab}	610616.9±65998.1^{ab}	0.131±0.014^{abc}	10019.5±467.4^{ab}
	15	TDZ	43.1±1.0^{ab}	40596.7±4384.7^{a}	413622.1±75527.6^{a}	0.147±0.011^{bcd}	8238.6±579.2^{a}
57	15	Control	42.2±0.5^{ab}	378621.4±15253.1^{efg}	2731985.8±48204.5^{gh}	0.138±0.005^{bcd}	42748.4±1760.9^{efgh}
	13	BA	34.8±0.7^{cde}	252826.0±13685.8^{def}	1620243.8±78722.6^{def}	0.159±0.005^{cde}	25481.8±1067.9^{cd}
	15	GA_3	39.0±0.3^{bc}	398073.5±19411.0^{efg}	2872085.6±65327.6^{h}	0.135±0.004^{bcd}	43891.2±1972.2^{fgh}
	15	GA_3+BA	33.1±0.3^{def}	233255.1±11981.3^{de}	1785066.2±73562.0^{ef}	0.132±0.004^{bcd}	28793.0±1843.8^{cdef}
	11	GA_3+TDZ	29.5±0.6^{efg}	256575.6±19974.1^{def}	1209502.1±103450.3^{bcde}	0.237±0.017^{defgh}	25743.6±1830.1^{cde}
	11	TDZ	32.5±0.8^{def}	155572.1±16778.6^{cd}	772975.6±65399.7^{abc}	0.199±0.014^{defg}	16346.9±887.9^{bc}
86	13	Control	38.2±0.6^{bcd}	526331.9±37478.6^{gh}	2901646.2±53980.4^{h}	0.177±0.010^{cdef}	58640.3±3749.9^{gh}
	12	BA	30.6±0.6^{ef}	962570.5±69017.8^{h}	2828428.5±83046.3^{h}	0.332±0.016^{gh}	74951.2±5786.7^{h}
	15	GA_3	39.9±0.7^{bc}	510246.9±40802.6^{fg}	2770450.6±75410.6^{gh}	0.179±0.012^{cde}	55914.7±44745.0^{gh}
	15	GA_3+BA	33.1±0.5^{def}	756560.6±55976.5^{gh}	2714538.0±81525.2^{gh}	0.266±0.013^{efgh}	51042.8±3037.4^{gh}
	11	GA_3+TDZ	23.9±0.7^{g}	645361.6±58066.3^{gh}	1668665.0±102229.8^{def}	0.382±0.024^{h}	40839.2±2588.3^{defg}
	9	TDZ	27.8±1.3^{fg}	506432.2±83039.3^{efg}	1476154.6±169083.5^{cdef}	0.324±0.022^{fgh}	40175.2±3527.3^{defg}
